# Effects of Clopidogrel and Proton Pump Inhibitors on Cardiovascular Events in Patients with Type 2 Diabetes Mellitus after Drug-Eluting Stent Implantation: A Nationwide Cohort Study

**DOI:** 10.1371/journal.pone.0135915

**Published:** 2015-08-27

**Authors:** Chi-Feng Hsieh, Weng-Foung Huang, Yi-Ting Chiang, Chun-Yen Chen

**Affiliations:** 1 Institute of Health and Welfare Policy, National Yang-Ming University, Taipei, Taiwan; 2 Division of Cardiology, Department of Internal Medicine, Mackay Memorial Hospital, Taipei, Taiwan; 3 Department of Medicine, Mackay Medical College, New Taipei City, Taiwan; University Hospital Medical Centre, GERMANY

## Abstract

**Objective:**

To investigate whether there is an increased risk of cardiac events in diabetic patients with a combined therapy of clopidogrel (CLO) and proton pump inhibitors (PPIs) after drug-eluting stent (DES) deployment.

**Methods:**

By using National Health Insurance Research Database, all patients who received CLO with or without PPI therapy within 90 days after undergoing DES (limus-eluting or paclitaxel-eluting stents) deployment were enrolled. Endpoints were acute coronary syndrome (ACS) and readmission for revascularization (percutaneous coronary intervention or coronary artery bypass graft surgery) after 3, 6, and 12 months.

**Results:**

A total of 6,603 diabetic patients received LESs (5,933 in the CLO subgroup and 670 in the CLO plus PPIs subgroup), and 3,202 patients received PESs (2,923 in the CLO subgroup and 279 in the CLO plus PPIs subgroup). The patients who received CLO plus PPIs were at higher risk of ACS than those receiving CLO within 1 year after DES deployment (LESs: 6-month hazard ratio [HR] = 1.63, and 1-year HR = 1.37; PESs: 3-month HR = 1.72). Patients with a history of ACS who received CLO plus PPIs were at higher risk of ACS after LES implantation (HR = 1.55) than those in the CLO group.

**Conclusion:**

In “real-world” diabetic patients with LES deployment, the combination of PPIs and CLO is associated with higher rates of ACS after 6 months and 1 year. Even after correction for confounding factors, concomitant PPI use remained an independent predictor of cardiac events, emphasizing the clinical importance of this drug—drug interaction.

## Introduction

Dual-antiplatelet therapy (DAPT) consisting of aspirin (acetylsalicylic acid [ASA]) and clopidogrel (CLO) is a cornerstone in the medical treatment of acute coronary syndrome (ACS) and after percutaneous coronary intervention (PCI). Compared with ASA alone, the combination of ASA and CLO was shown to significantly reduce the incidence of cardiovascular events after ACS. [1],[2],[3] In a recent comparison of bare-metal stents and drug-eluting stents (DESs), DESs reduce restenosis in every clinical situation and every type of lesion studied.[4] DESs have been in widespread use for more than a decade and are used in the majority of patients receiving intracoronary stents. Incomplete endothelialization, which makes DESs susceptible to late stent thrombosis, is frequently observed 6–12 months after the procedure.[5] DAPT was also considered to be essential after stent implantation to prevent early and late in-stent thrombosis.[6] However, a mono-prescription or dual-prescription antiplatelet treatment strategy is associated with an increased risk of gastrointestinal (GI) tract bleeding.[7] Proton pump inhibitors (PPIs) reduced antiplatelet-related GI tract bleeding among high-risk patients, including patients prescribed DAPT.[8] According to current US guidelines, PPIs are indicated with DAPT even in the absence of GI tract symptoms or in the presence of upper GI tract bleeding. [8],[9]

Several PPIs are metabolized by CYP2C19 and thus may interact with CLO metabolism.[7] Therefore, the use of concomitant PPIs could impede or prevent the metabolism of CLO to its active metabolites through competition for the same substrate, resulting in decreased activation of CLO, which leads to an increased risk of adverse cardiovascular events.[10],[11],[12] In Taiwan, concomitant PPI use was associated with an increased risk of rehospitalization and mortality.[13],[14] However, concurrent use of PPIs was not associated with increased rehospitalization for ACS or PCI in patients with previous ACS in Taiwan and was not associated with an increased risk of cardiovascular death, myocardial infarction (MI), or stroke in TRITON-TIMI 38.[15],[16] In Clopidogrel and the Optimization of Gastrointestinal Events (COGENT) Trial, over 30% of patients had diabetes, indicated that no significant increases in the risk of cardiovascular events with concomitant use of CLO and omeprazole.[17] However, the interaction between CLO and PPIs in diabetic patients with DES implantation in real-world practice has not been thoroughly investigated. To address this question, our study examined the impact of concomitant use of PPIs and CLO on cardiovascular outcomes of patients with limus-eluting stents (LESs) and paclitaxel-eluting stents (PESs) using a national medical database that covers 99.7% of the population in Taiwan.

## Materials and Methods

### Data Source

The data source for this study was the National Health Insurance Research Database (NHIRD), which covers 99.7% of the population (nearly 23 million people) in Taiwan. The NHIRD includes enrollment and claims files. The enrollment files contain individual subscription information and demographic factors, including sex, date of birth, type of beneficiaries, and location. The claims files contain comprehensive records of inpatient care, ambulatory care, pharmacy store, dental care, and Chinese medicine services, including date of service, International Classification of Diseases, 9th Revision, Clinical Modification (ICD-9-CM) diagnosis and procedure codes, claimed medical expenses, and the copayment amount for each encounter. This NHIRD dataset was provided by the National Health Research Institutes, and individual and provider identifiers have been encrypted in order to protect privacy and confidentiality. The study protocol was approved by the institutional review board of Taipei Veterans General Hospital (No 201006015IC).

### Study Group and Cohort Definition

We conducted a retrospective cohort study with a 6-year observation period (2006 to 2011). We identified 10,322 patients who (1) underwent LES or PES placement between January 1, 2007, and December 31, 2010; (2) had a diagnosis of type 2 diabetes mellitus (ICD-9-CM codes 250.x0 and 250.x2) before the first DES deployment; and (3) had at least one prescription for a hypoglycemic agent during a 1-year period prior to the first placement. Anti-diabetic medication can only be prescribed while patients fulfilled any one of following: 1) Fasting plasma glucose level ≥ 126 mg/dL; 2) Plasma glucose ≥ 200 mg/dL two hours after a 75 g oral glucose load as in a glucose tolerance test; 3) Symptoms of high blood sugar and casual plasma glucose ≥ 200 mg/dL or HbA_1_c≥ 6.5 under benefit package of NHI. We defined an individual’s index date as the date of discharge after the first DES implantation, and an individual was followed up for 1 year after the index date. Patients were excluded if (1) they did not have an ambulatory visit or receive a hypoglycemic agent within 90 days after the index date; (2) they experienced revascularization, ACS, or death within 7 days after the index date; (3) they underwent stent implantation within 1 year before the index date; or (4) they were implanted with both an LES and a PES concurrently at the index hospitalization. A total of 9,805 subjects were included in this analysis.

### Exposure

An individual’s exposure to CLO and PPIs within 90 days after the index date was identified using computer-based prescription claims; patients were classified into CLO (n = 8,856) or CLO plus PPIs (n = 949) groups. The algorithm to identify these drugs is described in the appendix.

### Outcomes and Covariates

Outcomes of this study consisted of two elements: time to event and a censoring indicator. Time to event represents the number of days from the index date to the date of the earliest of the following events: (1) the end of the observation and (2) the occurrence of target events, including revascularization (including PCI or coronary artery bypass graft [CABG] surgery) and ACS (ICD-9-CM codes 410.xx, 411.xx, and 414.9). The repeat revascularization was defined as PCI (ICD-9-CM codes 36.0–36.09) and CABG (ICD-9-CM codes 36.1–36.19). The observational period began at the cohort index date and continued until the first occurrence of any major adverse cardiac event or up to 1 year of follow up. Each patient had nine sets of outcomes given three endpoints (3 months, 6 months, and 1 year) and three target events. If the earliest event was the occurrence of a target event, and this record was not censored.

We considered the following covariates: age, sex, drug use after discharge (metformin, sulfonylurea, insulin, β-blocker, calcium-channel blocker, angiotensin-converting enzyme inhibitors or angiotensin II receptor blockers, lipid-lowering agents, and aspirin), and comorbidities during the year before the index date (congestive heart failure, ACS, renal disease, peripheral vascular disease, cerebrovascular disease, and chronic pulmonary disease), as defined by Romano et al.[18] We further adjusted for a history of CABG during the year before the index date and the intensity of the use of medical services during the index hospitalization, including the number of days spent in the hospital and the number of stents received.

### Statistical Analysis

We used the chi-square test to examine the association between two groups (CLO, and CLO plus PPIs) for categorical variables and *t* test for continuous variables. Multivariate-adjusted hazard ratios (HRs) were estimated using Cox proportional hazards models. We adjusted for potential confounders in the multivariable Cox models based on reported risk factors for cardiac events. The propensity score regression adjustment was also used to balance the distribution of confounders between the two groups (CLO group and CLO plus PPIs group) by summarizing all covariate information into a single probability and to simulate randomization.[19] Moreover, we further conducted a stratified analysis according to a history of ACS. All statistical tests were two sided, with an alpha level of 0.05, and the confidence intervals (CIs) were 95%. Data management and statistical analyses were performed using SAS version 9.2.

## Results

Of the 9,805 patients in our cohort, 6,603 patients received LESs (5,933 in the CLO group; 670 in the CLO plus PPIs group), and 3,202 patients received PESs (2,923 in the CLO group; 279 in the CLO plus PPIs group). The CLO plus PPIs subgroup was older than the CLO subgroup in both the LES and PES groups. In patients with LESs, compared with the CLO group, the CLO plus PPIs group had a higher proportion of patients with a history of CABG surgery and insulin use; a lower proportion of patients with metformin, sulfonylurea, and lipid-lowering agent use; lesser number of days in the hospital; and a lower Carlson comorbidity index. In patients with PESs, the CLO plus PPIs group had a higher proportion of patients with a history of chronic pulmonary disease and insulin use, a lower proportion of patients with metformin and lipid-lowering agent use, and lesser number of days spent in the hospital (Table 1).

**Table 1:** Characteristics of diabetes mellitus patients who had received DES implantation, stratified by medication taken within 3 months after DES implantation.

	LES (N = 6,603)			PES (N = 3,202)		
	CLO	CLO+PPI	*p*-value	CLO	CLO+PPI	*p*-value
	(N = 5,933)	(N = 670)		(N = 2,923)	(N = 279)	
Age, mean (SD), y	66.46±10.53	68.35±10.69	<0.001	65.61±10.37	67.77±9.62	<0.001
**Age group**						
<55 y	879 (14.82%)	87 (12.99%)	<0.001	465 (15.91%)	30 (10.75%)	0.01
55~64y	1,736 (29.26%)	150 (22.39%)		891 (30.48%)	69 (24.73%)	
65~74y	1,919 (32.34%)	228 (34.03%)		991 (33.90%)	112 (40.14%)	
≧75 y	1,399 (23.58%)	205 (30.60%)		576 (19.71%)	68 (24.37%)	
**Gender**						
Female	1,991 (33.56%)	244 (36.42%)	0.14	990 (33.87%)	110 (39.43%)	0.06
Male	3,942 (66.44%)	426 (63.58%)		1,933 (66.13%)	169 (60.57%)	
**Medical history in prior 1 y**						
Congestive heart failure	5,711 (96.26%)	653 (97.46%)	0.11	2,811 (96.17%)	273 (97.85%)	0.15
Acute coronary syndrome	1,858 (31.32%)	213 (31.79%)	0.80	1,019 (34.86%)	107 (38.35%)	0.24
Renal disease	5,476 (92.30%)	623 (92.99%)	0.53	2,698 (92.3%)	256 (91.76%)	0.74
Cerebrovascular disease	4,957 (83.55%)	568 (84.78%)	0.42	2,464 (84.30%)	236 (84.59%)	0.90
Peripheral vascular disease	3,574 (60.24%)	409 (61.04%)	0.69	1,741 (59.56%)	179 (64.16%)	0.13
Chronic pulmonary disease	4,677 (78.83%)	522 (77.91%)	0.58	2,259 (77.28%)	231 (82.80%)	0.03
Liver disease	1,580 (26.63%)	180 (26.87%)	0.90	748 (25.59%)	79 (28.32%)	0.32
Cancer	4,117 (69.39%)	483 (72.09%)	0.15	2,029 (69.41%)	192 (68.82%)	0.84
PCI	5,928 (99.92%)	670 (100.00%)	0.45	2,916 (99.76%)	279 (100.00%)	0.41
CABG surgery	58 (0.98%)	13 (1.94%)	0.02	21 (0.72%)	2 (0.72%)	1.00
**Characteristics of index hospitalization**						
Inpatient for ≤ 7 days	3,803 (64.1%)	335 (50.00%)	<0.001	1,993 (68.18%)	130 (46.59%)	<0.001
Stent no.>1	4,437 (74.79%)	518 (77.31%)	0.15	1,596 (54.6%)	169 (60.57%)	0.06
**Medication use during follow-up**						
Thiazolidinedione	942 (15.88%)	87 (12.99%)	0.05	463 (15.84%)	47 (16.85%)	0.66
Metformin	3,687 (62.14%)	338 (50.45%)	<0.001	1,885 (64.49%)	128 (45.88%)	<0.001
Sulfonylurea	3,650 (61.52%)	355 (52.99%)	<0.001	1,913 (65.45%)	175 (62.72%)	0.36
Insulin	1,263 (21.29%)	207 (30.90%)	<0.001	6,75 (23.09%)	98 (35.13%)	<0.001
DPP-4	994 (16.75%)	103 (15.37%)	0.36	2,34 (8.01%)	22 (7.89%)	0.94
β-blocker	4,348 (73.29%)	490 (73.13%)	0.93	2,159 (73.86%)	201 (72.04%)	0.51
Calcium channel blocker	4,424 (74.57%)	509 (75.97%)	0.43	2,162 (73.97%)	211 (75.63%)	0.54
ACEI/ARB	5,438 (91.66%)	606 (90.45%)	0.29	2,701 (92.41%)	257 (92.11%)	0.86
Lipid lowering agents	5,544 (93.44%)	612 (91.34%)	0.04	2,746 (93.94%)	252 (90.32%)	0.02
Antiplatelet agents						
Aspirin	4,235 (71.38%)	469 (70%)	0.45	2,125 (72.7%)	196 (70.25%)	0.38
Ticlopidine	334 (5.63%)	30 (4.48%)	0.22	156 (5.34%)	18 (6.45%)	0.43
Charlson comorbidity index	1.97±0.22	1.98±0.15	0.02	1.97±0.21	1.97±0.17	0.70

Abbreviation: LES, limus-eluting stent; PES, paclitaxel-eluting stent; CLO, clopidogrel; PPI, proton pump inhibitor; ACS, acute coronary syndrome; PCI: percutaneous coronary intervention; CABG: coronary artery bypass graft.

The crude incidence rate of readmission for revascularization was lower for the CLO subgroup than for the CLO plus PPIs subgroup (0.23 vs. 0.25 per person-year for PESs). The ACS incidence rate during follow up in the CLO and CLO plus PPI groups was 0.10 and 0.16 per person-year for LESs, respectively, and 0.12 and 0.17 per person-year for PESs, respectively. Across all events (readmission for revascularization or ACS), the CLO subgroup had a lower crude incidence rate of major adverse cardiac events than the CLO plus PPIs subgroup (Table 2).

**Table 2:** Patients follow-up, events, and incidence rate in patients with type 2 DM after DES implantation.

	LES (N = 6,603)		PES (N = 3,202)	
	CLO	CLO+PPI	CLO	CLO+PPI
**Readmission within 1 year**				
Mean follow-up time(SD),d	318.73±99.36	312.63±105.11	317.43±100.58	308.25±108.82
Total follow-up of time, person-days	1,891,022	209,465	927,844	86,002
**Revascularization or ACS Readmission**				
3 months	455 (7.67%)	58 (8.66%)	230 (7.87%)	25 (8.96%)
6 months	780 (13.15%)	103 (15.37%)	416 (14.23%)	46 (16.49%)
12 months	1,319 (22.23%)	161 (24.03%)	666 (22.78%)	73 (26.16%)
**Revascularization**				
Events(%), n	1,164 (19.62%)	127 (18.96%)	589 (20.15%)	58 (20.79%)
Incidence rate per person year	0.22	0.22	0.23	0.25
**ACS**				
Events(%), n	533 (8.98%)	92 (13.73%)	294 (10.06%)	41 (14.7%)
Incidence rate per person year	0.1	0.16	0.12	0.17
**Revascularization or ACS**				
Events(%), n	1,319 (22.23%)	161 (24.03%)	666 (22.78%)	73 (26.16%)
Incidence rate per person year	0.25	0.28	0.26	0.31

**Fig 1:**
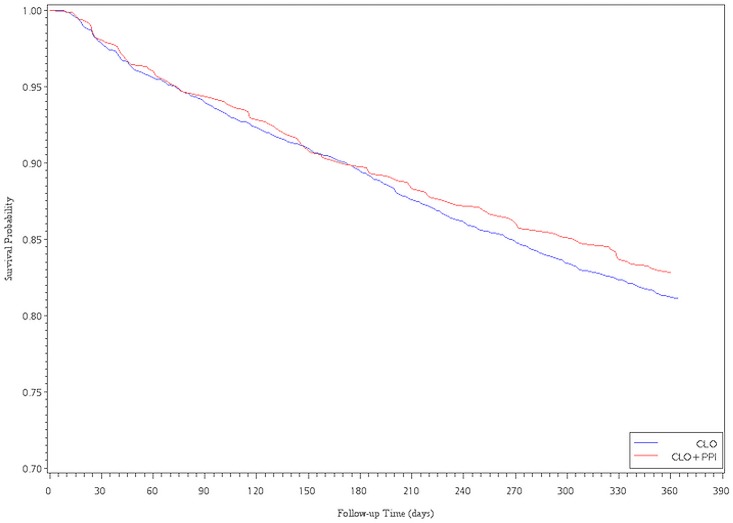
Survival analysis of revascularization versus exposure to ClO plus PPI and ClO after LES implantation.

**Fig 2:**
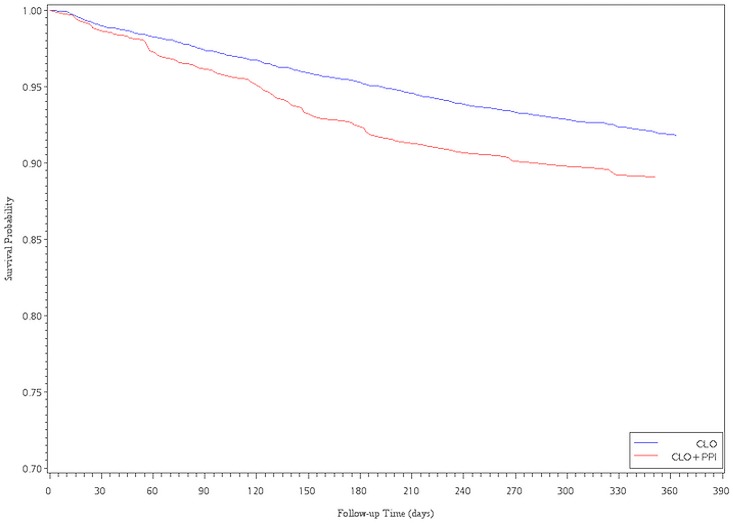
Survival analysis of ACS versus exposure to ClO plus PPI and ClO after LES implantation.

**Fig 3:**
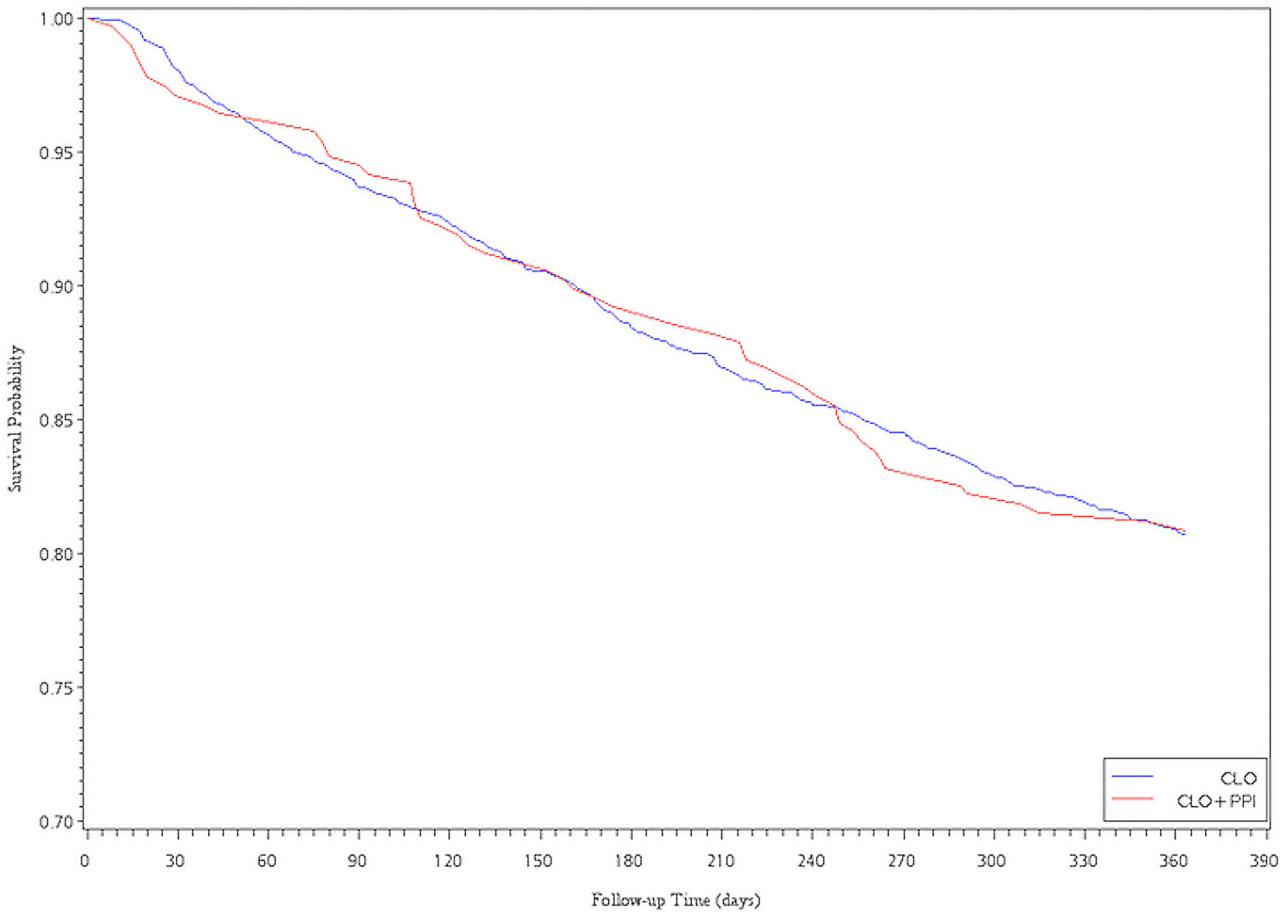
Survival analysis of revascularization versus exposure to ClO plus PPI and ClO after PES implantation.

**Fig 4:**
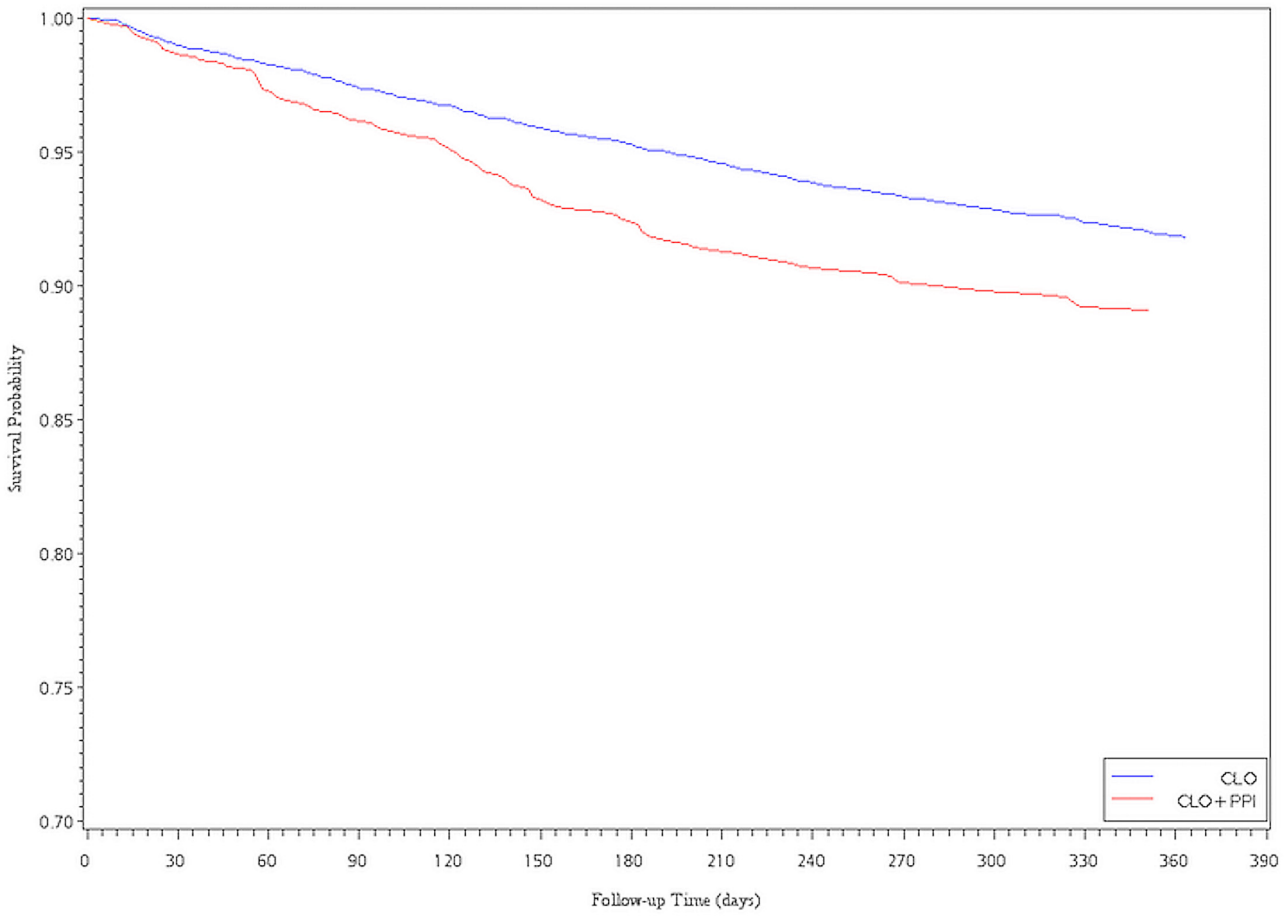
Survival analysis of ACS vs. exposure to ClO plus PPI and ClO after PES implantation.

The Cox proportional hazards analysis with propensity score adjustment showed an association between CLO plus PPI and a decreased risk of readmission for revascularization. However, there were no significant differences between the CLO and CLO plus PPIs subgroups, regardless of whether patients received LES or PES implantation (HR = 0.90, 95% CI, 0.75–1.09; HR = 1.00, 95% CI, 0.76–1.31, for LESs and PESs, respectively). After multivariate adjustment for ACS within 6 months and 1 year after the index date, the HR became significantly higher in the CLO plus PPIs group compared with that in the CLO group (HR = 1.63, 95%CI, 1.25–2.14; HR = 1.37, 95% CI, 1.09–1.71, for LESs). For PESs patients, the CLO plus PPIs subgroup had a significantly higher risk of ACS within 3 months (HR = 1.72, 95%CI, 1.02–2.89, for PESs) (Table 3). Of the 960 patients that were re-hospitalized due to ACS events, we also found that there were 146 (15.2%) patients taken CLO plus PPI, 634 (66.0%) patients taken CLO at the time point of re-intervention. Figs 1–4 show the survival analysis of readmission for revascularization or ACS associated with exposure to CLO plus PPIs and CLO after LES or PES deployment.

**Table 3:** Effect of exposure to clopidogrel versus clopidogrel plus PPI after DES implantation.

	LES (N = 6,603)			PES(N = 3,202)		
	HR*	95%CI	*p*-value	HR*	95%CI	*p*-value
**Revascularization**						
3 months	0.87	(0.63–1.21)	0.42	0.85	(0.51–1.42)	0.54
6 months	0.95	(0.75–1.21)	0.68	0.88	(0.61–1.27)	0.49
12 months	0.90	(0.75–1.09)	0.29	1.00	(0.76–1.31)	0.98
**ACS**						
3 months	1.45	(0.99–2.11)	0.06	1.72	(1.02–2.89)	0.04
6 months	1.63	(1.25–2.14)	<0.001	1.35	(0.89–2.04)	0.16
12 months	1.37	(1.09–1.71)	0.01	1.33	(0.95–1.87)	0.09
**Revascularization or ACS**						
3 months	1.03	(0.78–1.36)	0.84	1.00	(0.66–1.54)	0.99
6 months	1.09	(0.88–1.34)	0.44	1.04	(0.76–1.43)	0.79
12 months	1.01	(0.86–1.19)	0.90	1.11	(0.87–1.42)	0.42

*Adjusted for age, gender, drug use after discharge (metformin, sulfonylurea, insulin, β—blocker, calcium channel blocker, angiotensin-converting enzyme inhibitors/angiotensin II receptor blockers, lipid-lowering agents, and aspirin), comorbidities in 1 year before index date (congestive heart failure, myocardial infarction, renal disease, peripheral vascular disease, cerebrovascular disease, and chronic pulmonary disease), history of coronary artery bypass graft, number of days spent in hospital, number of stents, and propensity score.

Table 4 shows the stratification analysis by history of ACS. After propensity score adjustment for potential self-selection, we found that patients with a history of ACS who received CLO plus PPIs were at higher risk of ACS after LES implantation (HR = 1.55; 95% CI, 1.11–2.16) than those in the CLO group. There were no significant differences in the other subgroups.

**Table 4:** Stratified analysis of outcomes by history of ACS for clopidogrel versus clopidogrel added on PPI after DES implantation.

	Revascularization			ACS			Revascularization or ACS		
	HR*	95%CI	*p*-value	HR*	95%CI	p-value	HR*	95%CI	*p*-value
**LES**									
History of ACS (n = 2,071)	1.04	(0.77–1.41)	0.78	1.55	(1.11–2.16)	0.01	1.18	(0.91–1.53)	0.22
No history of ACS (n = 4,532)	0.83	(0.65–1.05)	0.13	1.24	(0.92–1.69)	0.16	0.92	(0.74–1.14)	0.42
**PES**									
History of ACS (n = 1,126)	0.94	(0.61–1.45)	0.77	1.31	(0.78–2.19)	0.31	1.06	(0.72–1.56)	0.78
No history of ACS (n = 2,076)	1.05	(0.73–1.50)	0.80	1.31	(0.84–2.05)	0.24	1.14	(0.82–1.59)	0.43

*Adjusted for age, gender, drug use after discharge (metformin, sulfonylurea, insulin, β—blocker, calcium channel blocker, angiotensin-converting enzyme inhibitors/angiotensin II receptor blockers, lipid-lowering agents, and aspirin), comorbidities in 1 year before index date (congestive heart failure, myocardial infarction, renal disease, peripheral vascular disease, cerebrovascular disease, and chronic pulmonary disease), history of coronary artery bypass graft, number of days spent in hospital, number of stents, and propensity score.

## Discussion

To the best of our knowledge, this study is the first to assess the association between the use of CLO plus PPIs and outcomes of patients with diabetes after different types of DES implantation. Our study explored the clinical effect of such potential interaction between CLO and PPIs by investigating the risk of adverse cardiovascular events in a nationwide, unselected population of CLO-treated diabetic patients after DES implantation. We found that the incidence of rehospitalization for ACS was higher in diabetic patients with DES deployment who were taking CLO plus PPIs, especially in patients with LES implantation, and that diabetic patients with previous MI undergoing LES deployment and taking CLO plus PPIs had a higher incidence of rehospitalization for ACS than those taking CLO alone. In a population-based nested case—control study, concomitant use of CLO with PPIs other than pantoprazole was associated with higher risk of reinfarction among patients receiving CLO following acute MI.[12] Another post hoc analysis of large registries also showed that patients who received CLO plus a PPI had a 93% higher risk of rehospitalization for MI than patients receiving CLO alone.[20] Our study had similar results, indicating that concomitant use of CLO and PPIs should be undertaken with caution in diabetic patients with DES implantation.

### Interaction between CLO and PPIs

When CLO is administered as a prodrug, 85% of it is inactivated by plasma esterases and the remaining 15% undergoes liver metabolism by the cytochrome P450 system to generate an active metabolite. The isoenzyme CYP2C19 plays an important role in CLO activation.[21] All anti-diabetic medication included in our study may not alter clopidogrel effect. [22] It has been shown that among CYP450 isoforms, a CYP2C19 loss-of-function variant is associated with a reduced response to CLO.[23] The prevalence of CYP2C19 loss-of-function alleles is much higher among Taiwanese than among other populations.[24] This might imply that a drug—drug interaction exists between CLO and PPIs. Matetzky et al reported that the lowest response to CLO in patients undergoing stenting for ST-elevated MI was associated with a 40% probability of a recurrent cardiovascular event within 6 months.[25] In patients undergoing PCI with DES placement, a low response to CLO was significantly associated with an increased risk of stent thrombosis.[26] After adjusting for risk factors such as age, sex, drug use after discharge, and comorbidity, concomitant use of PPI also predicted rehospitalization for ACS after DES deployment in our study. This suggests the possibility that a low response to CLO exists in patients with concomitant PPI use.

### Possible Explanations for the Observed Increased ACS Risk

In subgroups of patients with diabetes in randomized clinical trials, no significant difference in ACS was observed between LES and PES among diabetic patients.[27],[28] In the ISAR-DESIRE 2 trial, there were no significant differences in the MI rates between LES and PES among diabetic patients.[29] Although these studies demonstrated no significant difference in MI rates between LES and PES, the present study shows that MI rates are significantly increased in diabetic patients using CLO plus PPI after LES implantation. There are several possible explanations for the increased MI associated with concomitant treatment with CLO and PPIs after LES implantation. First, PPI therapy was independently associated with increased residual platelet reactivity and a higher frequency of high residual platelet reactivity (HRPR) and decreased the CLO inhibitory effect among patients with sirolimus-eluting stent implantation.[30] HRPR with CLO therapy has been associated with an increased risk of cardiovascular events after PCI.[31] Second, rapamycin can increase the activity of tissue factor, a key trigger for the coagulation cascade, in endothelial cells.[32] Jiang et al reported that platelet-endothelial adhesion was enhanced by rapamycin.[33] In contrast, paclitaxel significantly inhibited collagen-induced platelet aggregation in vitro.[34] The attenuated antiplatelet effect of CLO by PPI and increased platelet—endothelial adhesion by rapamycin might contribute to the increased MI rate with LES implantation. However, our study demonstrated that PPIs substantially affected the clinical outcomes of patients treated with LES. Prospective randomized studies are thus warranted to determine whether the findings from the present analysis of diabetic patients are generalizable to all stents eluting rapamycin analogs. In addition, further studies are required to understand the mechanisms underlying these findings so that more effective therapies can be developed for high-risk diabetic patients.

The present study has several limitations. First, the diagnoses in national health insurance claims primarily serve administrative-billing purposes and do not undergo verification for scientific purposes. However, subjects with PCI were ascertained by hospitalization for ACS and by stent implantation procedures, which were very reliable. Second, information regarding patient adherence or self-paid medications is not available. Although this could have lead to misclassification and biased the results towards a null effect, it would not have changed the clinically significant result regarding the increased risk of rehospitalization for ACS associated with CLO and PPI use. Third, death records were not available in the NHIRD, but we have captured important clinical outcomes of rehospitalization of ACS. Fourth, all the PPIs are available as prescription drugs in Taiwan. The possibility of misclassification of exposure to PPIs may be small in this study. Fifth, data regarding personal habits such as smoking, the severity of coronary artery disease, characteristics of coronary stenotic lesions (ex. instent stenosis, instent thrombosis or coronary lesions outside the initially implanted stent), and stent length and diameter were not available from this dataset. Also, NHIRD contained code of disease but did not contain important information like blood HbA1c, glucose levels, or platelet aggregometry. Therefore, we cannot provide information about these data correlate with clinical outcome or effect of PPI on the Adenosine diphosphate (ADP) induced platelet aggregation. In our study, PPI users were older, more female, and had more previous heart failure, ACS, cancer and liver disease, irrespective LES or PES, whereas non-PPI users took more often aspirin, ACEI and lipid lowering agents. Although propensity scoring and Charlson comorbidity index were included in the multivariate analysis, these factors could not be strongly excluded associated with worse outcomes. Therefore, further randomized study will be necessary to determine the effect of PPIs on CLO in different DESs. However, this study provides benchmark data for patients treated with different DESs and the inclusion of consecutive patients referred for PCI and stent implantation reflects a “real-world” situation and therefore strengthens our results.

### Clinical Implications

If a diabetic patient is at low risk of GI tract bleeding, it is best to avoid or decrease the dosage of PPIs used in combination with CLO, as the risk of negative interaction is greater than the risk of GI tract bleeding. Because there is no substantive evidence that PPIs attenuate the therapeutic effect of prasugrel or ticagrelor, these inhibitors might be considered if diabetic patients are at moderate or high risk of GI tract bleeding should it be necessary to use PPIs. Platelet function testing might be considered in specific high-risk patients (e.g. history of MI or high GI bleeding risk).

## Conclusion

We demonstrated that the adjunctive use of PPIs for the duration of CLO treatment increases the rate of rehospitalization due to ACS in diabetic patients after LES implantation. The use of PPIs in addition to CLO should be undertaken with caution in all diabetic patients with DES deployment, especially LES. We hope that our observation will stimulate future prospective trials to address this important clinical question.
